# Supplementary material for: The RNA m6A landscape during human oocyte-to-embryo transition
Source: EMBO J. 2025 Jun 4;44(14):4150–80. doi: 10.1038/s44318-025-00474-5 (PMC12264149; doi:10.1038/s44318-025-00474-5)
Supplement: Supplementary file 1 — Table EV1 [file 44318_2025_474_MOESM1_ESM.docx]

**Table EV1. Summary of picoMeRIP-seq data**

| **Stage/ Condition** | **Biological Replicate*** | **Number of oocytes or embryos or ES cells** | **Input/IP** | **Number of picoMeRIP-seq read pairs** | | | **Number of m6A peaks** |
| --- | --- | --- | --- | --- | --- | --- | --- |
|  |  |  |  | **Clean**** | **Uniquely aligned** | **Used***** |  |
| GV |  |  | Input | 42,924,961 | 36,304,176 | 4,052,085 | NA |
|  | Rep 1 | 1 | IP | 29,665,434 | 25,632,735 | 1,446,184 | 8,203 |
|  | Rep 2 | 1 | IP | 30,518,623 | 26,231,541 | 1,399,050 | 8,247 |
|  | Rep 3 | 1 | IP | 35,250,608 | 28,317,100 | 851,232 | 6,128 |
|  | Rep 4 | 1 | IP | 24,130,528 | 18,941,647 | 323,717 | 3,819 |
| MII |  |  | Input | 61,358,105 | 51,084,588 | 2,045,387 | NA |
|  | Rep 1 | 1 | IP | 48,002,822 | 36,265,959 | 1,075,113 | 6,933 |
|  | Rep 2 | 1 | IP | 36,487,012 | 27,960,477 | 1,235,335 | 6,591 |
|  | Rep 3 | 1 | IP | 34,444,029 | 27,410,800 | 404,676 | 3,073 |
| 1C |  |  | Input | 64,384,842 | 48,458,342 | 8,458,658 | NA |
|  | Rep 1 | 1 | IP | 35,666,765 | 11,218,916 | 1,480,000 | 6,003 |
|  | Rep 2 | 1 | IP | 25,753,822 | 14,069,279 | 1,150,926 | 4,798 |
|  | Rep 3 | 1 | IP | 33,895,416 | 17,849,639 | 977,245 | 4,820 |
| 2C |  |  | Input | 86,881,369 | 59,284,795 | 7,840,601 | NA |
|  | Rep 1 | 1 | IP | 135,514,575 | 35,654,772 | 595,858 | 5,044 |
|  | Rep 2 | 1 | IP | 100,766,865 | 62,664,670 | 1,551,107 | 9,715 |
| 8C |  |  | Input | 256,321,620 | 182,718,219 | 4,891,851 | NA |
|  | Rep 1 | 1 | IP | 89,462,171 | 16,406,170 | 250,241 | 5,276 |
|  | Rep 2 | 1 | IP | 98,271,413 | 37,576,934 | 581,192 | 6,921 |
| BLT |  |  | Input | 377,330,781 | 209,921,223 | 4,335,857 | NA |
|  | Rep 1 | 1 | IP | 30,359,197 | 19,633,163 | 1,005,658 | 10,002 |
|  | Rep 2 | 1 | IP | 27,232,527 | 17,804,895 | 1,098,262 | 9,200 |
|  | Rep 3 | 1 | IP | 29,877,789 | 20,772,092 | 841,075 | 10,678 |
|  |  |  |  |  |  |  |  |
| hESC |  |  | Input | 35,042,944 | 27,218,197 | 14,584,61  3 |  |
|  | Rep 1 | 1000 | IP | 27,410,539 | 23,507,344 | 7,177,080 | 29,923 |
|  | Rep 2 | 1000 | IP | 31,683,255 | 26,459,222 | 7,476,844 | 30,293 |
|  | Rep 1 | 100 | IP | 32,798,412 | 22,651,380 | 1,365,896 | 14,140 |
|  | Rep 2 | 100 | IP | 31,756,925 | 21,428,706 | 1,470,320 | 12,583 |
|  | Rep 1 | 10 | IP | 31,209,765 | 8,553,716 | 343,570 | 6,789 |
|  | Rep 2 | 10 | IP | 24,486,679 | 5,090,003 | 175,602 | 3,440 |
|  |  |  |  |  |  |  |  |
| hESC DMSO | Rep 1 | 5000 | Input | 49,248,117 | 34,995,362 | 8,534,336 |  |
|  |  |  | IP | 56,210,334 | 36,832,808 | 1,720,750 | 14,900 |
|  | Rep 2 | 5000 | Input | 53,771,721 | 37,476,533 | 8,326,300 |  |
|  |  |  | IP | 41,114,439 | 25,611,096 | 1,228,487 | 16,346 |
| hESC STM2457 | Rep 1 | 5000 | Input | 78,650,489 | 53,964,400 | 7,187,597 |  |
|  |  |  | IP | 64,819,810 | 12,153,636 | 602,631 | 4,625 |
|  | Rep 2 | 5000 | Input | 75,104,263 | 51,345,628 | 6,630,446 |  |
|  |  |  | IP | 44,849,121 | 12,662,680 | 529,523 | 3,933 |

* For each stage of human oocytes/embryos, two biological replicates, highlighted by yellow background, were selected for downstream analysis.

** Read pairs after quality control (by FastQC) and adapter removal (by Cutadapt)

*** Uniquely-aligned read pairs after removing PCR duplicates and ribosomal RNA-derived reads; and these reads were used for m^6^A peak calling by MACS2.
