# Supplementary material for: The RNA m6A landscape during human oocyte-to-embryo transition
Source: EMBO J. 2025 Jun 4;44(14):4150–80. doi: 10.1038/s44318-025-00474-5 (PMC12264149; doi:10.1038/s44318-025-00474-5)
Supplement: Supplementary file 3 — Dataset EV1–EV6 [file 44318_2025_474_MOESM3_ESM.zip › Li_et_al_Names_of_datasets.docx]

**Supplementary information**

**Dataset EV1**

Expression level and m6A status of all genes in GENCODE annotation library (v39).

**Dataset EV2**

Expression level and m6A status of homologous genes between human and mouse.

**Dataset EV3**

M-decay, Z-decay, ZGA and constantly expressed genes.

**Dataset EV4**

miRNA targeting of M-decay, Z-decay, ZGA and constantly expressed genes.

**Dataset EV5**

Translation efficiency of M-decay, Z-decay, ZGA and constantly expressed genes.

**Dataset EV6**

Expression level and m6A signal of retrotransposon loci/copies.
