# Supplementary material for: The RNA m6A landscape during human oocyte-to-embryo transition
Source: EMBO J. 2025 Jun 4;44(14):4150–80. doi: 10.1038/s44318-025-00474-5 (PMC12264149; doi:10.1038/s44318-025-00474-5)
Supplement: Supplementary file 4 — Expanded View Figures [file 44318_2025_474_MOESM4_ESM.pdf]

Expanded View Figures

Figure EV1. Performance evaluation of picoMeRIP-seq in human samples using hESCs and m^{6}A profiling in human oocytes and preimplantation embryos; along with the RNA expression levels of m^{6}A-related regulatory factors.

(**A**) Illustration of picoMeRIP-seq procedure performed on human oocytes and early embryos. The figure was created in BioRender. (**B**) Genome browser snapshots of two genes with m^{6}A enrichment in hESCs. (**C**) Transcriptome-wide correlation analyses of picoMeRIP-seq experiments (IP samples) among samples from 1000, 100 and 10 cells in hESCs. (**D**) Heatmap showing the fraction of m^{6}A-modified genes (identified in samples of 10 cells) over those identified in samples of 1000 and 100 cells, as well as over those from a previous study in hESCs (Batista et al, 2014). (**E**) Metagene profiles showing the enrichment of m^{6}A peaks along protein-coding genes in hESCs. (**F**) Consensus motifs identified within m^{6}A peaks in hESCs. (**G**) Principal component analysis (PCA) of transcriptome-wide m^{6}A signals. Multiple biological replicates for each stage were generated. (**H**) Number of m^{6}A peaks. For each stage, the averaged (mean) number across biological replicates 1 and 2 are shown. (**I**) Fraction of m^{6}A+ genes under different expression cutoffs. (**J**) Boxplot showing mRNA levels of m^{6}A associated proteins in human oocytes and early embryos. The data were downloaded from a previously published database EmAtlas (Zheng et al, 2023). The boxplots are arranged from top to bottom as follows: the maximal value, the 75th percentile, the median, the 25th percentile, and the minimal value. TB trophoblast.

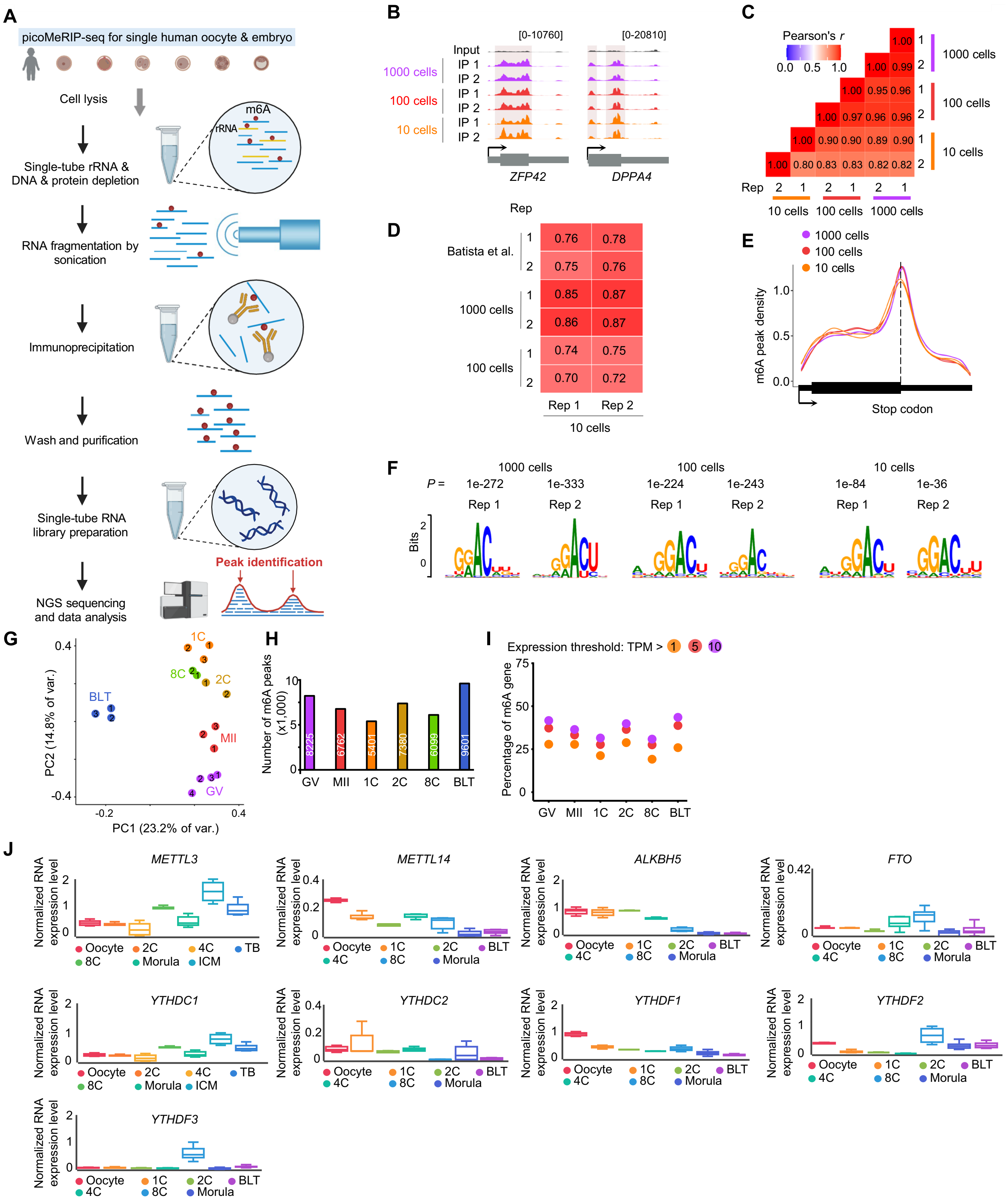
A
picoMeRIP-seq for single human oocyte & embryo
Cell lysis
m6A
rRNA
Single-tube rRNA & DNA & protein depletion
RNA fragmentation by sonication
Immunoprecipitation
Wash and purification
Single-tube RNA library preparation
Peak identification
NGS sequencing and data analysis
B
[0-10760]
[0-20810]
Input
IP 1
IP 2
1000 cells
100 cells
10 cells
ZFP42
DPPA4
C
Pearson's r
0.0 0.5 1.0
1000 cells
100 cells
10 cells
Rep
D
Rep
Batista et al.
1000 cells
100 cells
Rep 1
Rep 2
10 cells
E
1000 cells
100 cells
10 cells
m6A peak density
Stop codon
F
1000 cells
100 cells
10 cells
P =
1e-272
1e-333
1e-224
1e-243
1e-84
1e-36
Rep 1
Rep 2
Bits
G
BLT
1C
8C
2C
MII
GV
PC2 (14.8% of var.)
PC1 (23.2% of var.)
H
Number of m6A peaks (x1,000)
8225
6762
5401
7380
6099
9601
GV MII 1C 2C 8C BLT
I
Expression threshold: TPM > 1 5 10
Percentage of m6A gene
GV MII 1C 2C 8C BLT
J
METTL3
METTL14
ALKBH5
FTO
YTHDC1
YTHDC2
YTHDF1
YTHDF2
YTHDF3
Normalized RNA expression level
Oocyte
1C
2C
4C
8C
Morula
BLT
TB
ICM

 © The Author(s)

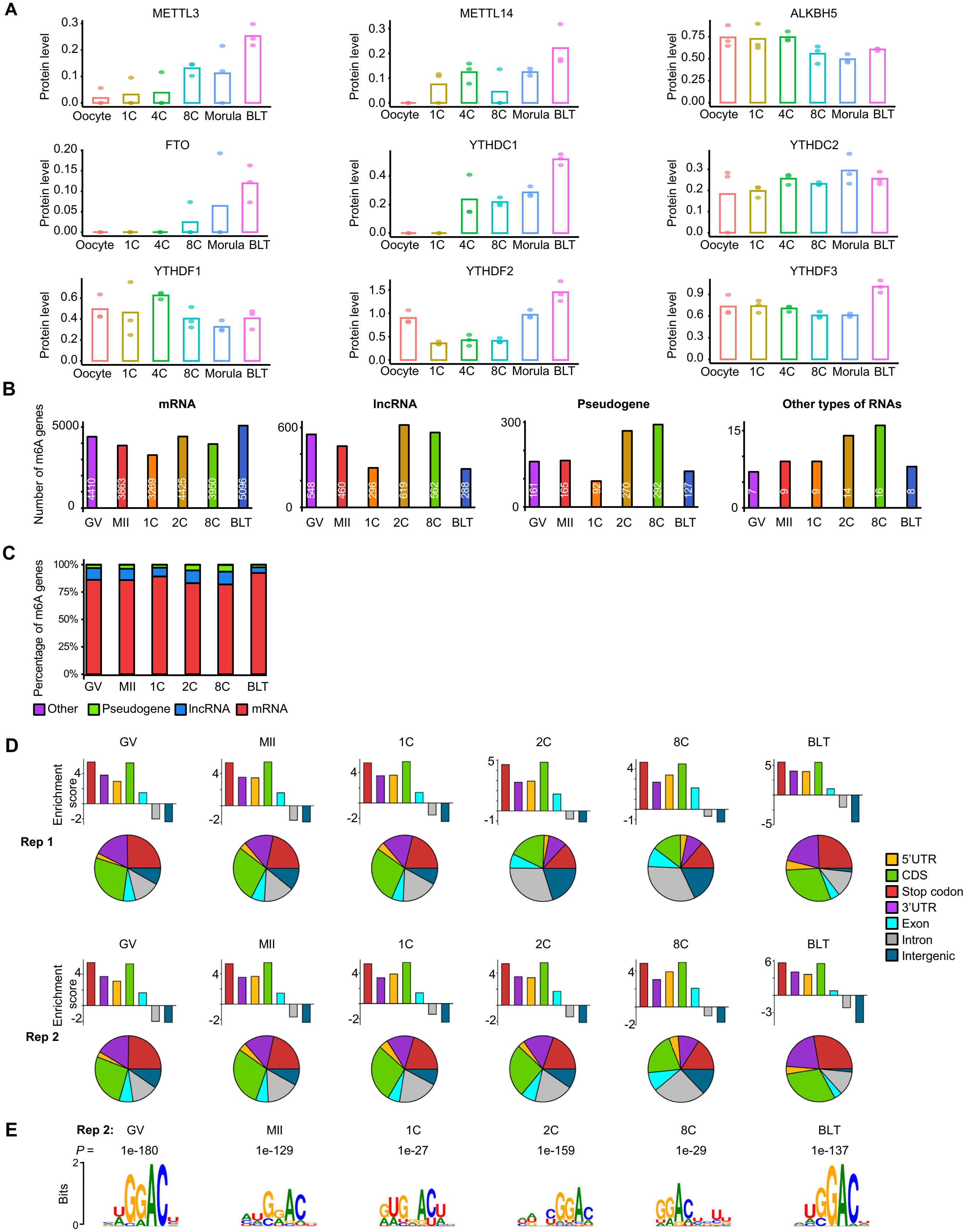
A
METTL3
METTL14
ALKBH5
FTO
YTHDC1
YTHDC2
YTHDF1
YTHDF2
YTHDF3
Protein level
Oocyte 1C 4C 8C Morula BLT
B
mRNA
lncRNA
Pseudogene
Other types of RNAs
Number of m6A genes
GV MII 1C 2C 8C BLT
4410 3863 3269 4425 3950 5096
548 460 296 619 562 288
161 165 92 270 292 127
7 9 9 14 16 8
C
Percentage of m6A genes
GV MII 1C 2C 8C BLT
Other Pseudogene lncRNA mRNA
D
Enrichment score
Rep 1
Rep 2
GV MII 1C 2C 8C BLT
5'UTR
CDS
Stop codon
3'UTR
Exon
Intron
Intergenic
E
Rep 2: GV MII 1C 2C 8C BLT
P = 1e-180 1e-129 1e-27 1e-159 1e-29 1e-137
Bits

Figure EV2. The distribution of m^{6}A peaks across different developmental stages; along with the protein expression levels of m^{6}A-related regulatory factors.

(**A**) Bar charts showing protein levels of m^{6}A associated proteins in human oocytes and early embryos. The data were downloaded from a previously published database (Zhu et al, 2025). The mean values were shown as the bar. (**B**) Number of m^{6}A+ genes for different types of genes. (**C**) Fraction of m^{6}A+ genes among different types of RNAs. (**D**) Top, pie charts show genomic annotation of m^{6}A peaks. Bottom, bar plots show the peak enrichment score, which was calculated as the $\log_2$ ratio of the observed over expected peak numbers. (**E**) Consensus motifs identified within m^{6}A peaks from biological replicate 2.

© The Author(s)

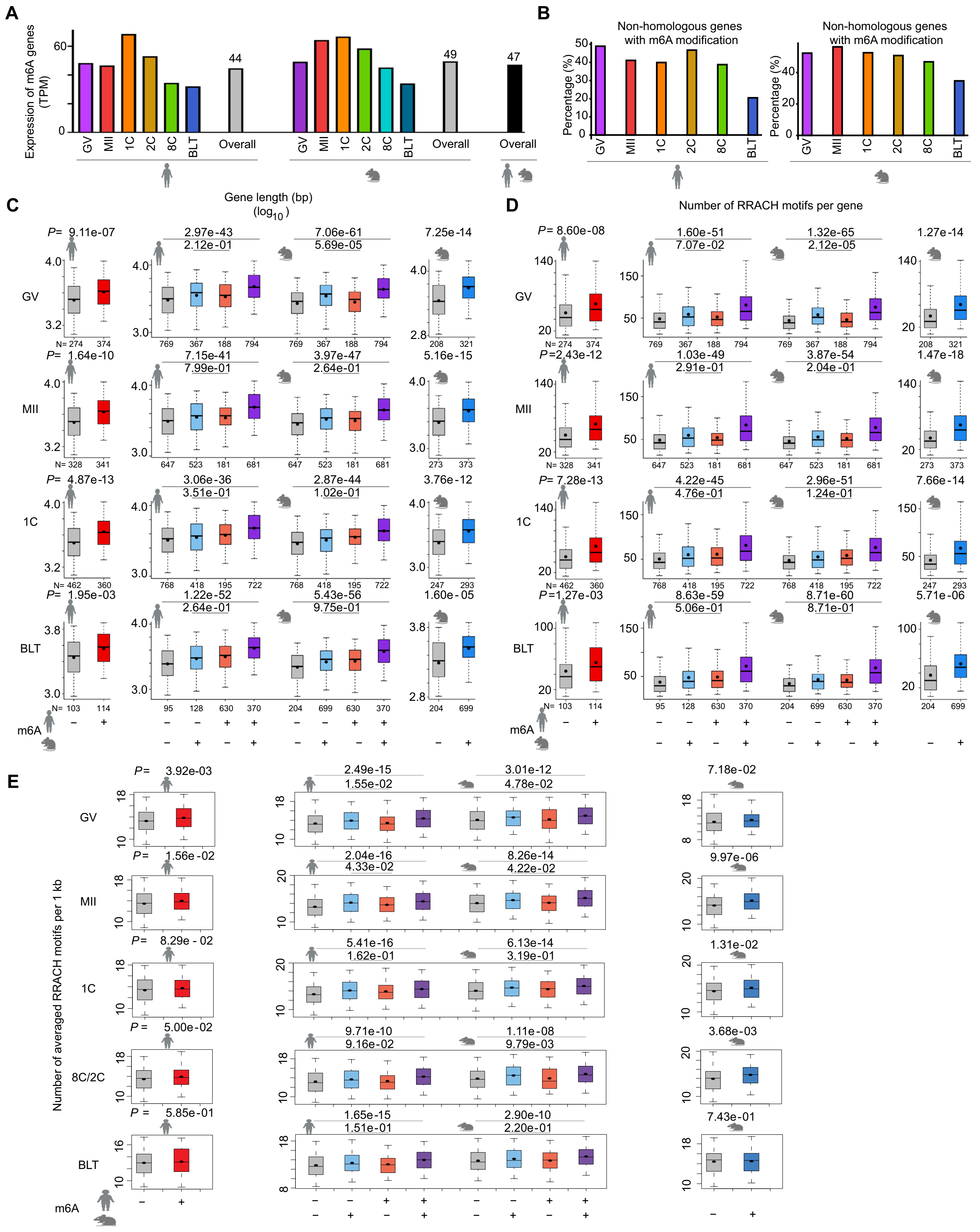
A
Expression of m6A genes (TPM)
GV MII 1C 2C 8C BLT Overall
44
49
47
B
Non-homologous genes with m6A modification
Percentage (%)
C
Gene length (bp) (log10)
D
Number of RRACH motifs per gene
GV
MII
1C
BLT
m6A
P= 9.11e-07
2.97e-43
2.12e-01
7.06e-61
5.69e-05
7.25e-14
P= 1.64e-10
7.15e-41
7.99e-01
3.97e-47
2.64e-01
5.16e-15
P= 4.87e-13
3.06e-36
3.51e-01
2.87e-44
1.02e-01
3.76e-12
P= 1.95e-03
1.22e-52
2.64e-01
5.43e-56
9.75e-01
1.60e-05
P= 8.60e-08
1.60e-51
7.07e-02
1.32e-65
2.12e-05
1.27e-14
P=2.43e-12
1.03e-49
2.91e-01
3.87e-54
2.04e-01
1.47e-18
P= 7.28e-13
4.22e-45
4.76e-01
2.96e-51
1.24e-01
7.66e-14
P=1.27e-03
8.63e-59
5.06e-01
8.71e-60
8.71e-01
5.71e-06
E
Number of averaged RRACH motifs per 1 kb
GV
MII
1C
8C/2C
BLT
m6A
P= 3.92e-03
2.49e-15
1.55e-02
3.01e-12
4.78e-02
7.18e-02
P= 1.56e-02
2.04e-16
4.33e-02
8.26e-14
4.22e-02
9.97e-06
P= 8.29e-02
5.41e-16
1.62e-01
6.13e-14
3.19e-01
1.31e-02
P = 5.00e-02
9.71e-10
9.16e-02
1.11e-08
9.79e-03
3.68e-03
P = 5.85e-01
1.65e-15
1.51e-01
2.90e-10
2.20e-01
7.43e-01

Figure EV3. Comparison of gene length and RRACH motif between m^{6}A modified and unmodified genes in human and mouse.

(**A**) Statistics of expression level of m^{6}A+ genes in human and mouse data. TPM, transcripts per million. (**B**) The ratios of non-homologous genes (TPM ≥ 50) with m^{6}A modification in human and mouse. (**C**, **D**) Comparison of gene lengths (**C**) and RRACH counts (**D**) between m^{6}A modified and unmodified genes for human- (left) and mouse- (right) specifically expressed genes, as well as human-mouse co-expressed genes (middle). The *P* values were calculated by the two-sided Wilcoxon rank-sum test. *N* number of genes used in the data analysis. (**E**) The average RRACH motif density after normalized per 1 kb length between m^{6}A modified and unmodified genes for human- (left) and mouse- (right) specifically expressed genes, as well as human-mouse co-expressed genes (middle). The *P* values were calculated by the two-sided Wilcoxon rank-sum test. *N* number of genes used in the data analysis. The boxplots in (**C–E**) were generated with five percentile-based whiskers, arranged from top to bottom as follows: the 95th percentile, the 75th percentile, the median, the 25th percentile, and the 5th percentile; and the mean value indicated by a dot.

 © The Author(s)

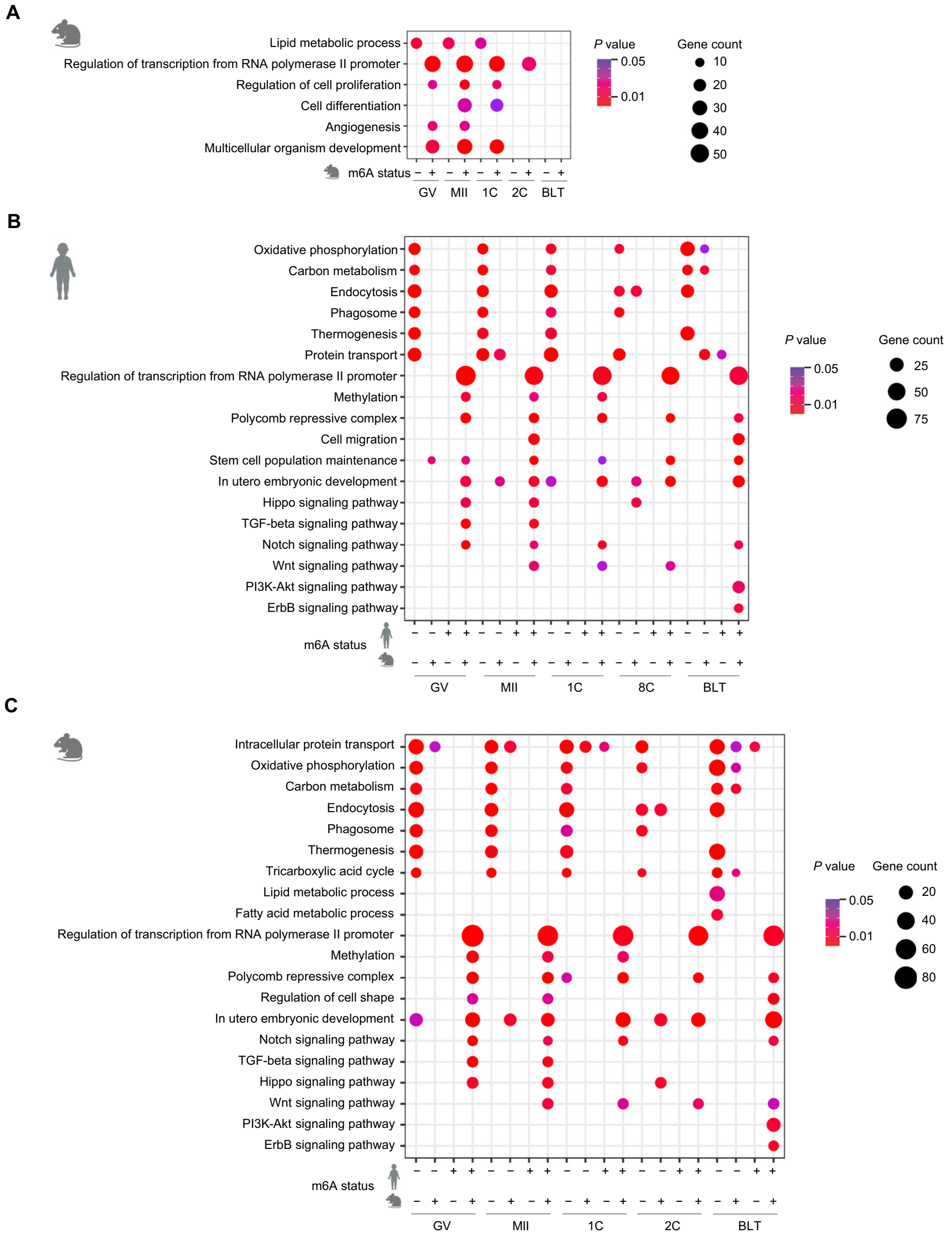

Figure EV4. GO analysis of m⁶A modified and unmodified genes for mouse-specifically expressed genes and human-mouse co-expressed genes.

(**A–C**) GO analysis for m⁶A +/− genes for mouse specifically expressed genes (**A**), for human genes that were co-expressed in mouse (**B**), and for mouse genes that were co-expressed in human (**C**). Fisher's exact test was used to calculate the one-sided *P* values.

© The Author(s)

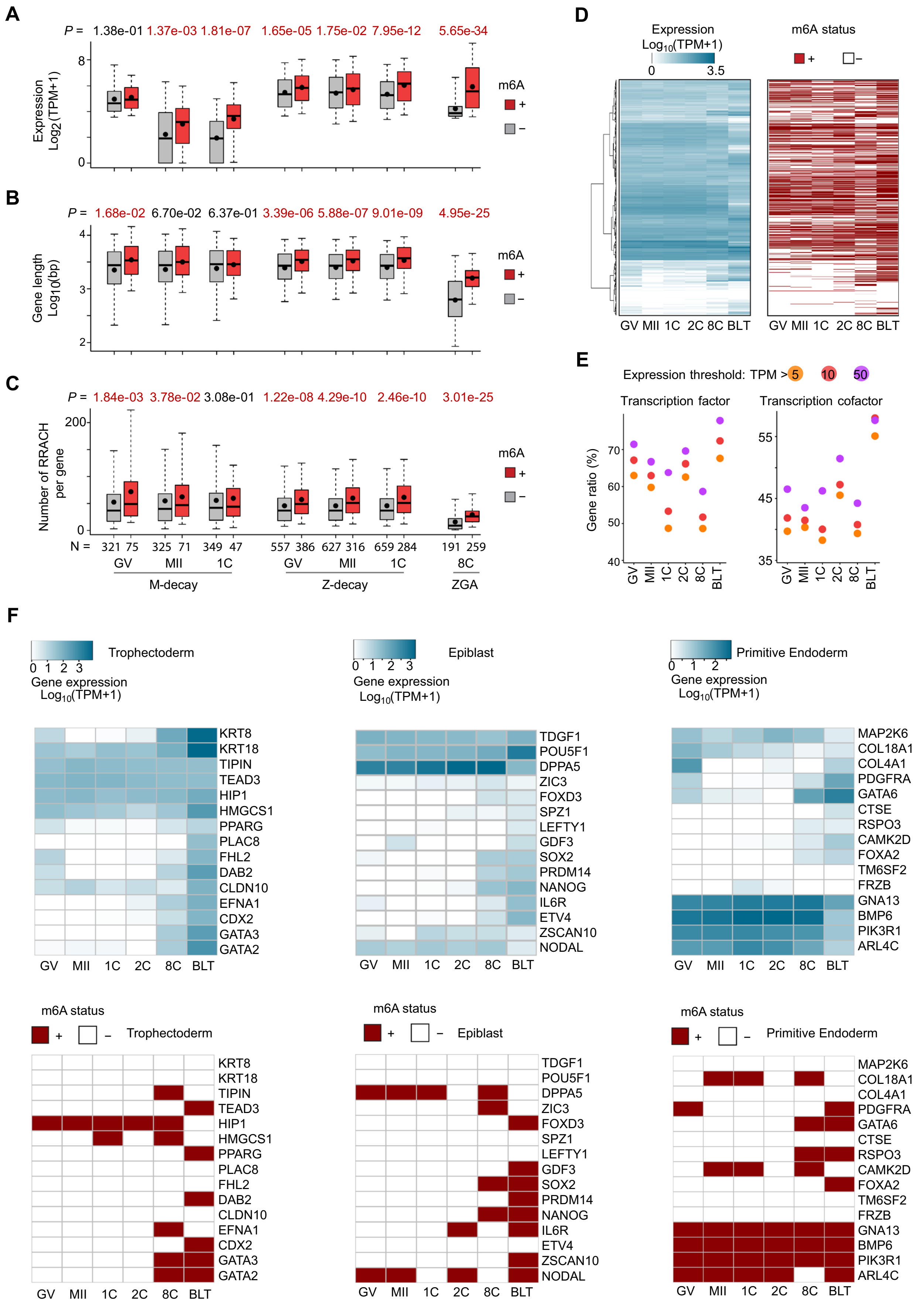
A
P = 1.38e-01 1.37e-03 1.81e-07 1.65e-05 1.75e-02 7.95e-12 5.65e-34
Expression Log2(TPM+1)
m6A + −
B
P = 1.68e-02 6.70e-02 6.37e-01 3.39e-06 5.88e-07 9.01e-09 4.95e-25
Gene length Log10(bp)
C
P = 1.84e-03 3.78e-02 3.08e-01 1.22e-08 4.29e-10 2.46e-10 3.01e-25
Number of RRACH per gene
N = 321 75 325 71 349 47 557 386 627 316 659 284 191 259
GV MII 1C GV MII 1C 8C
M-decay Z-decay ZGA
D
Expression Log10(TPM+1)
m6A status
GV MII 1C 2C 8C BLT
E
Expression threshold: TPM > 5 10 50
Transcription factor
Transcription cofactor
Gene ratio (%)
GV MII 1C 2C 8C BLT
F
Trophectoderm
Epiblast
Primitive Endoderm
Gene expression Log10(TPM+1)
KRT8 KRT18 TIPIN TEAD3 HIP1 HMGCS1 PPARG PLAC8 FHL2 DAB2 CLDN10 EFNA1 CDX2 GATA3 GATA2
TDGF1 POU5F1 DPPA5 ZIC3 FOXD3 SPZ1 LEFTY1 GDF3 SOX2 PRDM14 NANOG IL6R ETV4 ZSCAN10 NODAL
MAP2K6 COL18A1 COL4A1 PDGFRA GATA6 CTSE RSPO3 CAMK2D FOXA2 TM6SF2 FRZB GNA13 BMP6 PIK3R1 ARL4C
m6A status + −

 © The Author(s)

Figure EV5. Comparison of m^{6}A-modified and unmodified genes in terms of M-decay, Z-decay, and ZGA categories; along with the expression levels and m^{6}A modification status of transcription factors and cofactors.

(**A–C**) Comparison of expression levels (**A**), gene lengths (**B**), and RRACH counts (**C**) between m^{6}A modified and unmodified genes for M-decay, Z-decay and ZGA genes. The *P* values were calculated by the two-sided Wilcoxon rank-sum test. The boxplots were generated with five percentile-based whiskers, arranged from top to bottom as follows: the 95th percentile, the 75th percentile, the median, the 25th percentile, and the 5th percentile; and the mean value indicated by a dot. *N* number of genes used in the data analysis. (**D**) Expression (left) and m^{6}A status (right) dynamics of transcription cofactor coding genes. (**E**) Proportion of m^{6}A+ transcription factors (left) and transcription cofactors (right) coding genes at each stage with different expression thresholds. (**F**) Expression and m^{6}A marking status of genes essential for the lineage specification events in human early embryos. Top: gene expression, down: m^{6}A status.

© The Author(s)

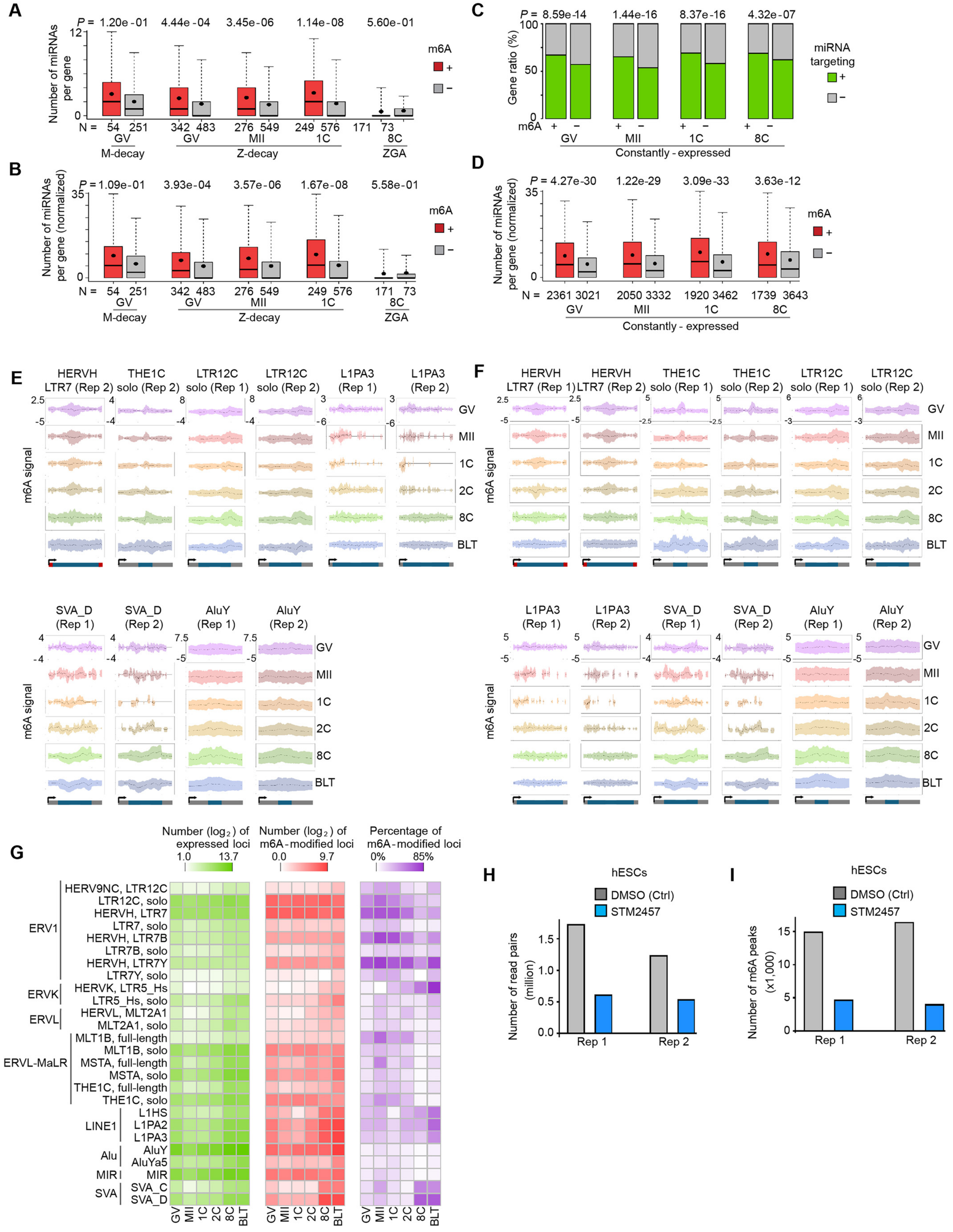
A
P = 1.20e-01 4.44e-04 3.45e-06 1.14e-08 5.60e-01
Number of miRNAs per gene
m6A
+
−
N = 54 251 342 483 276 549 249 576 171 73
GV GV MII 1C 8C
M-decay Z-decay ZGA
B
P = 1.09e-01 3.93e-04 3.57e-06 1.67e-08 5.58e-01
Number of miRNAs per gene (normalized)
N = 54 251 342 483 276 549 249 576 171 73
C
P = 8.59e-14 1.44e-16 8.37e-16 4.32e-07
Gene ratio (%)
miRNA targeting
m6A
GV MII 1C 8C
Constantly - expressed
D
P = 4.27e-30 1.22e-29 3.09e-33 3.63e-12
N = 2361 3021 2050 3332 1920 3462 1739 3643
E
HERVH LTR7 (Rep 2)
THE1C solo (Rep 2)
LTR12C solo (Rep 1)
LTR12C solo (Rep 2)
L1PA3 (Rep 1)
L1PA3 (Rep 2)
m6A signal
GV MII 1C 2C 8C BLT
SVA_D (Rep 1)
SVA_D (Rep 2)
AluY (Rep 1)
AluY (Rep 2)
F
HERVH LTR7 (Rep 1)
HERVH LTR7 (Rep 2)
THE1C solo (Rep 1)
THE1C solo (Rep 2)
LTR12C solo (Rep 1)
LTR12C solo (Rep 2)
L1PA3 (Rep 1)
L1PA3 (Rep 2)
SVA_D (Rep 1)
SVA_D (Rep 2)
AluY (Rep 1)
AluY (Rep 2)
G
Number (log2) of expressed loci 1.0 13.7
Number (log2) of m6A-modified loci 0.0 9.7
Percentage of m6A-modified loci 0% 85%
ERV1
HERV9NC, LTR12C
LTR12C, solo
HERVH, LTR7
LTR7, solo
HERVH, LTR7B
LTR7B, solo
HERVH, LTR7Y
LTR7Y, solo
ERVK
HERVK, LTR5_Hs
LTR5_Hs, solo
ERVL
HERVL, MLT2A1
MLT2A1, solo
ERVL-MaLR
MLT1B, full-length
MLT1B, solo
MSTA, full-length
MSTA, solo
THE1C, full-length
THE1C, solo
LINE1
L1HS
L1PA2
L1PA3
Alu
AluY
AluYa5
MIR
MIR
SVA
SVA_C
SVA_D
GV MII 1C 2C 8C BLT
H
hESCs
DMSO (Ctrl)
STM2457
Number of read pairs (million)
Rep 1 Rep 2
I
hESCs
DMSO (Ctrl)
STM2457
Number of m6A peaks (x1,000)
Rep 1 Rep 2

 © The Author(s)

Figure EV6. Comparison of miRNA targeting and translation efficiency between m^{6}A modified and unmodified genes for M-decay, Z-decay and ZGA genes, as well as for constantly expressed genes; along with the analysis of m^{6}A enrichment on retrotransposon-derived RNAs.

(**A**) Comparison of the count of miRNAs targeting m^{6}A modified versus unmodified mRNA genes for M-decay, Z-decay and ZGA genes. The *P* values were calculated by the two-sided Wilcoxon rank-sum test. *N* number of genes used in the data analysis. (**B**) Comparison of the normalized count of miRNAs targeting m^{6}A modified versus unmodified mRNA genes for M-decay, Z-decay and ZGA genes. See "Methods" for the calculation of normalized mRNA counts. The *P* values were calculated by the two-sided Wilcoxon rank-sum test. *N* number of genes used in the data analysis. (**C**) Comparison of the proportion of mRNA genes targeted by miRNAs between m^{6}A modified and unmodified genes for constantly expressed genes. The *P* values were calculated by the one-sided Fisher's exact test. (**D**) Comparison of the normalized count of miRNAs targeting m^{6}A modified versus unmodified mRNA genes for constantly expressed genes. See "Methods" for the calculation of normalized mRNA counts. The *P* values were calculated by the two-sided Wilcoxon rank-sum test. *N* number of genes used in the data analysis. (**E**) Distribution of m^{6}A signals along the full-length sequences of the representative retrotransposon subfamilies, based on unique assignment strategy. The mean (represented by the central black line) and standard deviation (represented by the band around the black line) across all loci were plotted. These plots are based on uniquely aligned reads. (**F**) Distribution of m^{6}A signals along the full-length sequences of the representative retrotransposon subfamilies, based on a random assignment strategy. The mean (represented by the central black line) and standard deviation (represented by the band around the black line) across all loci were plotted. These plots are based on a random assignment strategy for those multiply aligned reads. (**G**) Heatmaps showing the number of expressed loci (left), the number of those with m^{6}A among the expressed loci (middle), and the percentage of loci with m^{6}A (right) for the representative retrotransposon subfamilies. These results are based on a random assignment strategy for those multiply aligned reads. (**H**) Comparison of the number of processed sequencing reads between control (DMSO) and treatment (STM2457) groups in human ESCs. (**I**) Comparison of the number of m^{6}A peaks between control (DMSO) and treatment (STM2457) groups in human ESCs. The boxplots in (**A**, **B**, **D**) were generated with five percentile-based whiskers, arranged from top to bottom as follows: the 95th percentile, the 75th percentile, the median, the 25th percentile, and the 5th percentile; and the mean value indicated by a dot.

© The Author(s)

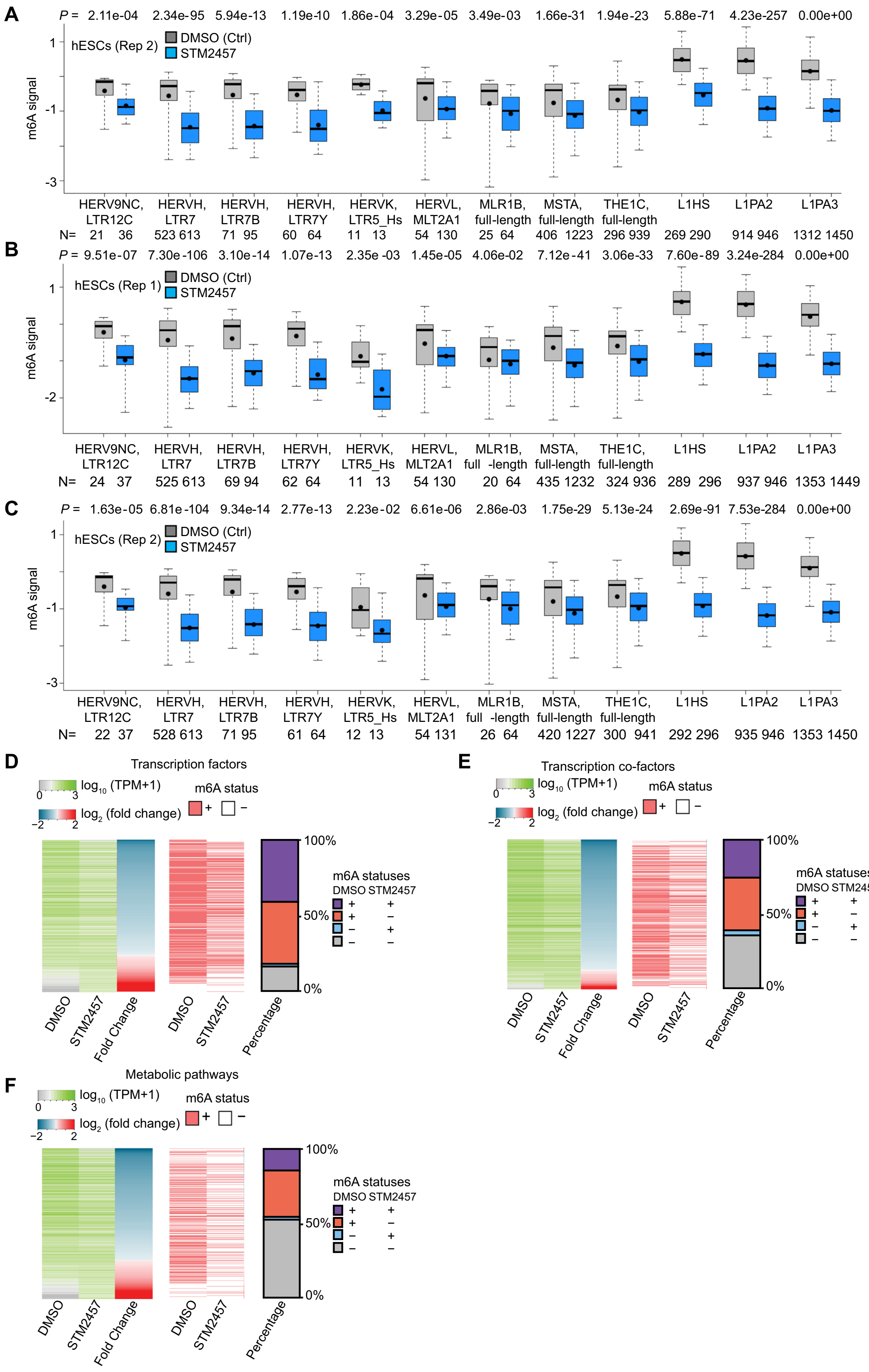
A
P = 2.11e-04 2.34e-95 5.94e-13 1.19e-10 1.86e-04 3.29e-05 3.49e-03 1.66e-31 1.94e-23 5.88e-71 4.23e-257 0.00e+00
hESCs (Rep 2)
DMSO (Ctrl)
STM2457
m6A signal
HERV9NC, LTR12C HERVH, LTR7 HERVH, LTR7B HERVH, LTR7Y HERVK, LTR5_Hs HERVL, MLT2A1 MLR1B, full-length MSTA, full-length THE1C, full-length L1HS L1PA2 L1PA3
N= 21 36 523 613 71 95 60 64 11 13 54 130 25 64 406 1223 296 939 269 290 914 946 1312 1450
B
P = 9.51e-07 7.30e-106 3.10e-14 1.07e-13 2.35e-03 1.45e-05 4.06e-02 7.12e-41 3.06e-33 7.60e-89 3.24e-284 0.00e+00
hESCs (Rep 1)
DMSO (Ctrl)
STM2457
m6A signal
N= 24 37 525 613 69 94 62 64 11 13 54 130 20 64 435 1232 324 936 289 296 937 946 1353 1449
C
P = 1.63e-05 6.81e-104 9.34e-14 2.77e-13 2.23e-02 6.61e-06 2.86e-03 1.75e-29 5.13e-24 2.69e-91 7.53e-284 0.00e+00
hESCs (Rep 2)
DMSO (Ctrl)
STM2457
m6A signal
N= 22 37 528 613 71 95 61 64 12 13 54 131 26 64 420 1227 300 941 292 296 935 946 1353 1450
D
Transcription factors
log10 (TPM+1)
log2 (fold change)
m6A status
m6A statuses
DMSO STM2457
DMSO STM2457 Fold Change DMSO STM2457 Percentage
E
Transcription co-factors
F
Metabolic pathways

© The Author(s)

Figure EV7. Analysis of m^{6}A profile changes after STM2457 treatment in hESC.

(**A**) Comparison of the m^{6}A signal on representative retrotransposon subfamilies between DMSO and STM2457 groups in hESCs (based on uniquely aligned reads from biological replicate 2). The *P* values were calculated by the two-sided Wilcoxon rank-sum test. *N* number of repeat loci used in the data analysis. (**B**, **C**) Comparison of the m^{6}A signal on representative retrotransposon subfamilies between DMSO and STM2457 groups in hESCs (based on a random assignment strategy for those multiply aligned reads; see "Methods"). The (**B**) is biological replicate 1; and (**C**) is biological replicate 2. The *P* values were calculated by the two-sided Wilcoxon rank-sum test. *N* number of repeat loci used in the data analysis. (**D–F**) The expression level and fold change (left), m^{6}A status (middle) and percentage (right) of transcription factor (**D**), transcription cofactors (**E**) and metabolic pathways (**F**) mRNAs. The boxplots in (**A–C**) were generated with five percentile-based whiskers, arranged from top to bottom as follows: the 95th percentile, the 75th percentile, the median, the 25th percentile, and the 5th percentile; and the mean value indicated by a dot.

© The Author(s)
